# Differences in risk factors between patterns of recurrence in patients after curative resection for advanced gastric carcinoma

**DOI:** 10.1186/1477-7819-11-98

**Published:** 2013-05-17

**Authors:** Yoshitsugu Nakanishi, Masanori Ohara, Hiromitsu Domen, Toshiaki Shichinohe, Satoshi Hirano, Masanori Ishizaka

**Affiliations:** 1 Department of Surgery, National Hospital Organization, Hakodate Hospital, 18-16 Kawahara-cho, Hakodate 041-8512, Japan; 2 Second Department of Gastroenterological Surgery, Hokkaido University Graduate School of Medicine, North 15, West 7 Kita-ku, Sapporo 060-8638, Japan

**Keywords:** Gastric carcinoma, Patterns of recurrence, Prognosis, Risk factor

## Abstract

**Background:**

Recurrence patterns in patients who have undergone curative gastrectomy for advanced gastric carcinoma can be classified as peritoneal, hematogenous, or lymphatic. The aim of this study was to clarify differences in risk factors between these different types of recurrence pattern.

**Methods:**

Postoperative courses, including sites of recurrence and periods between surgery and recurrence, of patients who had undergone curative gastrectomy for advanced gastric carcinoma (more than pT2 invasion) were surveyed in detail. Clinicopathological factors were examined as potential independent risk factors for each recurrence pattern, based on recurrence-free survival, using multivariate analysis.

**Results:**

Multivariate analysis identified depth of tumor invasion (pT4 vs. pT2/3; hazard ratio (HR), 7.05; *P* < 0.001), number of lymph node metastases (pN2/3 vs. pN0/1; HR, 4.02; *P* = 0.001), and histological differentiation (G3/4 vs. G1/2; HR, 2.22; *P* = 0.041) as independent risk factors for peritoneal metastasis. The number of lymph node metastases (HR, 26.21; *P* < 0.001) and venous vessel invasion (HR, 5.09; *P* = 0.001) were identified as independent risk factors for hematogenous metastasis. The number of lymph node metastases (HR, 6.00; *P* = 0.007) and depth of tumor invasion (HR, 4.70; *P* = 0.023) were identified as independent risk factors for lymphatic metastasis.

**Conclusions:**

This study clarified differences in risk factors between various patterns of recurrence. Careful examination of risk factors could help prevent oversight of recurrences and improve detection of recurrences during follow-up. The number of lymph node metastases represents an independent risk factor for all three patterns of recurrence; thus, patients with multiple lymph node metastases warrant particular attention.

## Background

Even after performing curative surgical resection, death from recurrence is frequent among patients with advanced gastric carcinoma. However, early detection of recurrence sites is sometimes difficult. One reason for this is that recurrence can show various patterns. Recurrence patterns in patients who have undergone curative surgical resection for advanced gastric carcinoma can be classified as peritoneal, hematogenous, or lymphatic metastases. Clarification of the differences in risk factors between these patterns of recurrence may be helpful in postoperative follow-up to ensure that recurrences are not missed and to allow additional therapy, including chemo- or radiotherapy, to be initiated early in the recurrence phase.

The aim of this study, therefore, was to clarify differences in risk factors between these three recurrence patterns among patients who had undergone curative resection for advanced gastric carcinoma.

## Methods

### Patients

Patients with synchronous primary neoplasms of other organs or who had undergone neoadjuvant chemotherapy were excluded from the study. A total of 132 patients (87 men, 45 women) who had undergone surgical curative resection and had been pathologically diagnosed with advanced gastric carcinoma (defined as carcinoma extending more deeply than the muscularis propria) between April 1999 and December 2011 at the National Hospital Organization at Hakodate Hospital, Hakodate, Japan, were registered in the study. All these patients showed negative results on intra-operative peritoneal cytology. The median age at the time of surgery was 69 years (range, 30 to 92 years). Surgical procedures for these patients involved total gastrectomy for 53 patients, distal gastrectomy for 70, proximal gastrectomy for 6, and pancreaticoduodenectomy for 3. The extent of lymph node dissection was D2 level in 71 patients and below D2 in 61, according to the 2010 Japanese gastric cancer treatment guidelines [1]. Adjuvant treatment after surgical resection was administered at the discretion of the individual surgeon. A total of 61 patients (including 3 of 19 patients in stage 1, 15 of 51 in stage 2, and 43 of 61 in stage 3 according to the *TNM Classification of Malignant Tumors*[2]) received oral administration of S-1 or UFT for approximately 1 year, or until side effects became too strong to tolerate.

### Postoperative follow-up

Most patients received regular follow-up sessions every 3 months. At each visit, a clinical examination, hematological analysis (including tumor marker assays for carcinoembryonic antigen and carbohydrate antigen 19-9), and chest and abdominal radiography were performed. Digestive endoscopy was performed annually. Follow-up ended in March 2012. The median survival period for all patients was 32 months (range, 1 to 157 months).

Computed tomography of the abdomen was performed every 6 months or on suspicion of clinical recurrence, including when an increase in tumor markers above pathological levels was seen. Bone scintigraphy was used for suspected bone metastasis. If an intestinal obstruction was not improved by long tube insertion, the patient was examined for peritoneal dissemination and underwent surgery if necessary.

### Clinicopathological factors

This study examined eight clinicopathological factors as candidate risk factors for recurrence after curative resection of advanced gastric carcinoma: extent of the primary tumor (pT2/3 vs. pT4); number of metastatic lymph nodes (pN0/1 vs. pN2/3); histopathological grading (G1/2, including papillary carcinoma, vs. G3/4, including signet ring cell carcinoma, mucinous adenocarcinoma, in accordance with the *TNM Classification of Malignant Tumors*[2]); venous invasion; lymphatic vessel invasion; sex; age (<70 years vs. ≥70 years); and extent of systematic lymphadenectomy (D2 or less than D2, according to Japanese gastric cancer treatment guidelines 2010 [1]). In this study, performance of adjuvant chemotherapy was not examined as a candidate risk factor of recurrence, because this factor correlated with other factors (pT4 and pN2/3).

### Prognostic factors for overall survival

Risk factors for overall survival were examined using univariate and multivariate analysis to compare them for each pattern of recurrence.

### Examinations of risk factors according to patterns of recurrence

The type of recurrence was classified on the basis of imaging studies or intra-operative and biopsy findings in patients who underwent re-operation. The incidence of recurrence depends on the time from surgical resection. We therefore examined for risk factors associated with the time of recurrence-free survival (RFS):

1. RFS was defined as the interval between completion of surgery and recurrence.

2. For patients with two or three recurrence patterns detected asynchronously, RFS for all recurrence patterns was defined as the interval between surgery and the first recurrence pattern.

3. Patients with two or three recurrence patterns detected simultaneously were classified as showing all the recurrence patterns detected.

4. In an examination for one pattern of recurrence, data from patients with only the other recurrence patterns were censored as of the date of occurrence of the other recurrence patterns.

5. Data for patients who did not experience recurrence were censored as of the date of the final observation.

6. Data for patients who died without recurrence were censored as of the date of death.

### Statistical analysis

Survival curves were constructed using the Kaplan-Meier method and differences in overall survival and RFS on univariate analysis were evaluated using the log-rank test. The Cox proportional hazards model was used to perform multivariate analysis. All tests were two-sided; values of *P* < 0.05 were considered statistically significant.

## Results

### Recurrence patterns

Among the 132 patients who underwent curative resection for advanced gastric carcinoma, 66 were alive without recurrence and 6 were alive with recurrence of gastric carcinoma, as of March 2012, while 21 patients had died of other diseases without evident recurrence of gastric carcinoma and 39 had died of recurrent gastric carcinoma.

**Figure 1:**
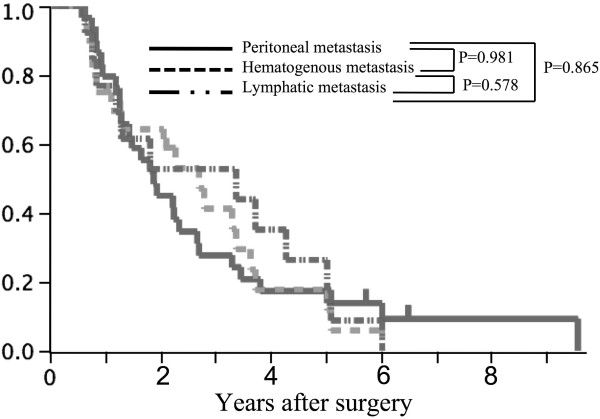
Overall survival curves of patient with recurrence in three recurrence patterns.

Of the 45 patterns of recurrence, peritoneal-only, hematogenous-only, lymphatic-only, all three patterns combined, hematogenous with lymphatic, peritoneal with hematogenous, and peritoneal with lymphatic patterns were seen in 21, 8, 2, 4, 5, 2, and 3 patients, respectively. Overall survival curves after surgical resection with the three recurrence patterns are shown in Figure 1. The median overall survival period for peritoneal, hematogenous, and lymphatic metastasis patterns was 22.6 months (range, 7 to 115 months), 32.5 months (8 to 72 months), and 40.5 months (8 to 72 months), respectively. No statistical difference was seen between the three recurrence patterns (*P* = 0.939).

### Prognostic factors in overall survival

The impacts of clinicopathological variables on overall survival in all 132 patients are shown in Table 1. Histological differentiation, depth of tumor invasion, number of lymph node metastases, lymphatic vessel invasion, and venous vessel invasion were identified as prognostic factors for overall survival on univariate analysis (*P* = 0.020, *P* < 0.001, *P* < 0.001, *P* = 0.016, and *P* = 0.026, respectively). On multivariate analysis, histological differentiation, depth of primary tumor invasion, and number of lymph node metastases were identified as independent factors affecting overall survival (*P* = 0.006, *P* = 0.040, and *P* < 0.001, respectively).

**Table 1:** Univariate and multivariate analyses of overall survival

**Variable**	***n***	**Survival rate at 5 years (%)**	**Univariate *P***	**Multivariate *P *relative risk (95% confidence interval)**
Age (years)			0.104	
<70	74	61.7		
≥70	58	45.2		
Sex			0.165	
Male	87	58.8		
Female	45	47.0		
Lymphadenectomy			0.647	
<D2	61	56.2		
D2	71	54.8		
Histological differentiation			0.002	0.006
G1 or G2	58	70.7		1
G3 or G4	74	41.2		2.15 (1.23 to 3.85)
Depth of tumor invasion			<0.001	0.040
pT2 or pT3	64	77.2		1
pT4	68	35.6		1.85 (1.03 to 3.47)
Number of lymph node metastases			<0.001	<0.001
pN0 or pN1	74	72.8		1
pN2 or pN3	58	36.5		2.71 (1.53 to 4.98)
Lymphatic invasion			0.016	
Negative	45	70.7		
Positive	87	48.1		
Venous invasion			0.026	
Negative	84	62.7		
Positive	48	39.4		

### Risk factors for recurrence patterns

#### Peritoneal metastasis

The median RFS for the 30 patients with peritoneal metastasis was 14.5 months (range, 3.4 to 64.2 months). The impacts of clinicopathological variables on the RFS of peritoneal recurrence are shown in Table 2. On univariate analysis, histological differentiation, depth of tumor invasion, and number of lymph node metastases were identified as risk factors for peritoneal metastasis (*P* = 0.022, *P* < 0.001, and *P* < 0.001, respectively). On multivariate analysis, histological differentiation, depth of tumor invasion, and number of lymph node metastases represented independent risk factors associated with peritoneal metastasis (*P* = 0.041, *P* < 0.001, and *P* = 0.001, respectively).

**Table 2:** Univariate and multivariate analyses of recurrence-free survival for peritoneal metastasis

**Variables**	***n***	**Cumulative recurrence rate at 5 years (%)**	**Univariate *P***	**Multivariate *P *relative risk (95% confidence interval)**
Age (years)			0.460	
<70	74	30.6		
≥70	58	30.0		
Sex			0.126	
Male	87	23.6		
Female	45	48.7		
Lymphadenectomy			0.595	
<D2	61	29.0		
D2	71	33.0		
Histological differentiation			0.022	0.041
G1 or G2	58	20.4		1
G3 or G4	74	42.3		2.22 (1.03 to 5.17)
Depth of tumor invasion			<0.001	<0.001
pT2 or pT3	64	8.0		1
pT4	68	53.6		7.05 (2.42 to 30.05)
Number of lymph node metastases			<0.001	0.001
pN0 or pN1	74	13.2		1
pN2 or pN3	58	53.7		4.02 (1.03 to 5.17)
Lymphatic invasion			0.120	
Negative	45	23.1		
Positive	87	36.0		
Venous invasion			0.441	
Negative	84	29.1		
Positive	48	34.6		

#### Hematogenous metastasis

Recurrence sites in the 19 patients with hematogenous recurrence were the liver in ten patients (52.6%), bone in four (21.1%), pleura in three (15.8%), lungs in three (15.8%), brain in two (10.5%), and intramural residual stomach, non-resected stump or site of anastomosis, in two (10.5%). Some patients had recurrence in more than one site.

The median RFS of the 19 patients with peritoneal metastasis was 14.2 months (range, 2.1 to 59.8 months). The impacts of clinicopathological variables on RFS for hematogenous metastasis are shown in Table 3. On univariate analysis, depth of tumor invasion, number of lymph node metastases, lymphatic vessel invasion, and venous invasion were identified as risk factors for hematogenous metastasis (*P* = 0.009, *P* < 0.001, *P* = 0.004, and *P* < 0.001, respectively). On multivariate analysis, the number of lymph node metastases and venous vessel invasion represented independent risk factors for hematogenous metastasis (*P* < 0.001 and *P* = 0.001, respectively).

**Table 3:** Univariate and multivariate analyses of recurrence-free survival for hematogenous metastasis

**Variables**	***n***	**Cumulative recurrence rate at 5-year (%)**	**Univariate *P***	**Multivariate *P *relative risk (95% confidence interval)**
Age (years)			0.309	
<70	74	27.1		
≥70	58	13.5		
Sex			0.505	
Male	87	20.2		
Female	45	28.3		
Lymphadenectomy			0.234	
<D2	61	20.9		
D2	71	25.1		
Histological differentiation			0.094	
G1 or G2	72	14.5		
G3 or G4	59	31.2		
Depth of tumor invasion			0.009	0.708
pT2 or pT3	64	13.0		1
pT4	68	33.1		1.23 (0.43 to 4.08)
Number of lymph node metastases			<0.001	<0.001
pN0 or pN1	74	2.0		1
pN2 or pN3	58	47.7		26.21 (3.66 to 581.73)
Lymphatic invasion			0.004	0.982
Negative	45	3.3		1
Positive	87	32.5		1.03 (0.14 to 22.26)
Venous invasion			<0.001	0.001
Negative	84	12.9		1
Positive	48	49.3		5.09 (1.89 to 14.87)

#### Lymphatic metastasis

The median RFS for the 14 patients with lymphatic metastasis (including 3 patients with lymphangiosis carcinomatosa) was 15.3 months (range, 4.2 to 59.8 months). The impacts of clinicopathological variables on RFS for lymphatic metastasis are shown in Table 4. On univariate analysis, depth of tumor invasion and number of lymph node metastases were identified as risk factors for lymphatic metastasis (*P* = 0.001, and *P* < 0.001, respectively). On multivariate analysis, depth of tumor invasion and number of lymph node metastases represented independent risk factors for lymphatic metastasis (*P* = 0.023 and *P* = 0.007, respectively).

**Table 4:** Univariate and multivariate analyses of recurrence-free survival for lymphatic metastasis

**Variables**	***n***	**Cumulative recurrence rate at 5-year (%)**	**Univariate *P***	**Multivariate *P *relative risk (95% confidence interval)**
Age (years)			0.949	
<70	74	21.2		
≥70	58	12.3		
Sex			0.609	
Male	87	16.9		
Female	45	23.0		
Lymphadenectomy			0.492	
<D2	61	20.4		
D2	71	18.5		
Histological differentiation			0.074	
G1 or G2	58	14.6		
G3 or G4	74	27.9		
Depth of tumor invasion			0.001	0.023
pT2 or pT3	64	7.7		1
pT4	68	31.2		4.70 (1.21 to 31.28)
Number of lymph node metastases			< 0.001	0.007
pN0 or pN1	74	5.0		1
pN2 or pN3	58	38.1		6.00 (1.56 to 39.83)
Lymphatic invasion			0.073	
Negative	45	8.4		
Positive	87	24.8		
Venous invasion			0.122	
Negative	84	14.9		
Positive	48	35.3		

## Discussion

This study examined differences in risk factors between various patterns of recurrence in patients who underwent surgical curative resections for advanced gastric carcinoma. As a result, independent risk factors for each recurrence pattern were identified as follows. For peritoneal metastasis, depth of tumor invasion, number of lymph node metastases, and histological differentiation were identified. For hematogenous metastasis, number of lymph node metastases, and venous vessel invasion were identified. For lymphatic metastasis, depth of tumor invasion, and number of lymph node metastases were identified.

Seeding of cancer cells into the abdominal cavity represents the first step in peritoneal metastasis. This means that pT4 can reasonably be considered an independent risk factor for peritoneal metastasis, as previously reported [3-6]. Histological differentiation was also detected as a risk factor for peritoneal metastasis in some reports [4,6-9]. Although some reports have described lymph node metastasis as an independent risk factor for peritoneal metastasis, as in our result [4,5,10-12], the role of lymph node metastasis in peritoneal metastasis has been unclear. However, because peritoneal recurrence occurred in patients with cancer limited to the gastric mucosa or submucosa but with lymph node metastasis, invasion of the lymphatic system by cancer cells has been suggested as the mechanism underlying peritoneal recurrence [13,14]. Moreover, injury to the lymphatic system during operative procedures in patients with highly extensive metastatic lymph nodes may allow the spread of viable cancer cells into the peritoneal cavity [12].

The first step in hematogenous metastasis is invasion of cancer cells into the lumen of the venous circulation. Our finding of vessel invasion as an independent factor for hematogenous metastasis is reasonable. The same result has been reported from other institutions [15,16]. However, vessel invasion is not incorporated as a factor in the Union for International Cancer Control (UICC) staging criteria or the Japanese classification of gastric carcinoma. Attention should thus be given to hematogenous recurrence in patients showing vessel invasion, even if the tumor stage is otherwise comparatively low. Conversely, the number of lymph node metastases might be an independent risk factor for hematogenous metastasis because of the connection of lymphatic channels to the systemic circulation via the thoracic duct. Noguchi *et al*. [16] reported venous invasion and lymph node metastasis as risk factors for liver metastasis. Kodera *et al*. [17] reported lymph node metastasis as a risk factor for bone metastasis.

With regard to lymphatic metastasis, the number of lymph node metastases and depth of tumor invasion represented independent risk factors. In this study, however, lymphatic vessel invasion was not identified as a risk factor for lymphatic metastasis, perhaps for the following reasons. First, cancer cells flow through lymphatic vessels to distant vessels. Cancer cells invading lymphatic vessels are therefore sometimes not detected in resected specimens. Second, lymphatic vessels are sometimes difficult to distinguish from the venous vasculature. In addition, some lymphatic vessels are thought to be destroyed by invasion of cancer cells, so pathologists cannot always detect lymphatic vessel invasions correctly. Third, if the number of cancer cells invading lymphatic vessels is small, the invasion might not be reflected in the patient prognosis. In addition, quantifying the grade of lymphatic vessel invasion objectively is difficult. However, the number of lymph node metastases reflects outflow of cancer cells into lymphatic vessels. In our study, the number of lymph node metastases was stratified into N0/N1 or N2/N3. This stratification of lymph node metastasis may be considered to reflect the amount of cancer cells invading lymphatic networks better than the presence or absence of lymphatic vessel invasion.

As with our result, some reports have described the number of lymph node metastases as a risk factor for lymphatic metastasis [12,14]. The identification of pT4 as an independent factor for lymphatic metastasis might reflect cancer cell invasion into the entire subserosal layer through the abundant lymphatic vessels.

The status of lymph node metastasis has been identified as the most important prognostic factor in patients undergoing gastrectomy [3,4,7,18-21]. This is reflected in the fact that the number of lymph node metastases represented an independent prognostic factor for all three patterns of recurrence in the present study. As mentioned previously, the number of lymph node metastases might reflect the amount of cancer cells in lymphatic channels in the peritoneum and both the greater and lesser omentum. Preoperative neoadjuvant chemotherapy for patients with a strong indication of lymph node metastases might therefore be acceptable to reduce seeding of cancer cells into the abdominal cavity as the result of surgical procedures. However, no randomized studies have yet addressed the survival benefits of this approach [1]. Randomized controlled trials of neoadjuvant therapy for patients with lymph node metastasis are thus needed to clarify means of achieving better prognosis in patients undergoing curative resection.

## Conclusions

Risk factors for recurrence after curative gastrectomy for advanced gastric carcinoma differ between patterns of recurrence. By paying more attention to the specific risk factors of recurrence present in patients, the likelihood of missing sites of recurrence could be decreased and recurrences identified earlier. This would allow appropriate treatment to be initiated more quickly for patients with recurrence. In addition, the status of lymph node metastasis contributed to all patterns of recurrence, even peritoneal metastasis. For patients in whom lymph node metastasis is suspected preoperatively, neoadjuvant therapy might be utilized to achieve better treatment outcomes.

## Abbreviations

HR: Hazard ratio; RFS: Recurrence-free survival; UICC: Union for International Cancer Control.

## Competing interests

The authors declare that they have no competing interests.

## Authors’ contributions

MO, HD, and MI carried out surgical resections. MO, TS and SH revised the manuscript for important intellecutual content. YN carried out surgical resections and collected clinical data and designed this study and drafted the manuscript. All authors read and approved the final manuscript.
